# Interventional radiology exposure and preparedness: Insights from private-sector radiologists without interventional subspecialisation and recent graduates in South Africa

**DOI:** 10.4102/sajr.v30i1.3560

**Published:** 2026-10-26

**Authors:** Peter Zuidewind, Jacques Janse van Rensburg

**Affiliations:** 1 Department of Diagnostic Radiology, Faculty of Health Sciences, University of the Free State, Bloemfontein, South Africa

**Keywords:** radiology education, interventional radiology, medical education, curriculum development, private practice, South Africa, specialist training, clinical competence

## Abstract

**Background:**

The national radiology curriculum in South Africa lacks procedural and content-specific detail. Most recent graduates are expected to find work in the private sector, but little is known about the spectrum of interventional procedures performed in this sector by radiologists without interventional subspecialist training.

**Objectives:**

To assess interventional radiology exposure and preparedness for private practice among recent graduates in South Africa who do not pursue interventional subspecialisation.

**Method:**

One questionnaire was distributed to private-practice radiologists without interventional subspecialisation, assessing the range of interventional procedures they perform, and another to recent radiology graduates, evaluating their self-reported competencies acquired during training.

**Results:**

Hepatic or pancreatic biopsy, renal biopsy, lumbar puncture and abdominal abscess drainage were frequently performed procedures in private practice. Recent graduates reported the highest exposure rates and self-reported competence in these procedures. Lower frequencies and self-reported competence were reported for biliary procedures, percutaneous nephrostomies and vascular procedures.

**Conclusion:**

This exploratory, respondent-based study suggests partial alignment between South African radiology registrar training and the interventional procedures commonly encountered in private practice by radiologists without interventional subspecialisation. These preliminary interpretations support the case for a revised national curriculum that will improve consistency across training institutions.

**Contribution:**

Heterogeneity in registrar training in interventional radiology highlights the need for a national South African curriculum that provides adequate procedural and content-specific detail.

## Introduction

Interventional radiology (IR) is a rapidly advancing and increasingly important subspecialty of radiology. Despite this, the South African diagnostic radiology curriculum does not specify the interventional competencies expected of diagnostic radiologists upon completion of their registrar training.^1^ Recent research has shown that South African radiology registrars feel the need for more IR exposure, with only a few reporting they had a structured, institution-specific IR curriculum.^2^

It is anecdotally known that most newly qualified radiologists in South Africa find work in the private sector. Still, which IR procedures they are expected to perform in private practice, if they do not have IR subspecialist training, is not known. Understanding these expectations will help align training with the demands of private practice. To the author’s knowledge, no similar prior study has been performed in South Africa. This study aimed to evaluate whether radiology training in South Africa adequately prepares recent graduates to meet private-practice expectations, specifically regarding IR procedures in the absence of IR subspecialist training.

## Methods

### Study design, participants and sampling

The study was descriptive, cross-sectional and quantitative in nature. Two questionnaires were distributed via e-mail to prospective participants, and data collection was web-based. The Radiological Society of South Africa (RSSA) facilitated dissemination of the questionnaires by distributing them to their members through their mailing list. After initial distribution by the RSSA, private radiology practices were contacted to offer participation to eligible candidates. Academic institutions were also contacted, asking them to forward the link to recent radiology graduates. Data collection was conducted between April 2024 and July 2024.

Questionnaire 1 (Q1) was completed by radiologists trained in South Africa and currently working in private practice. Radiologists who performed work both in the private and public sectors were included, but were only asked about their work in the private sector. Respondents with subspecialist qualifications in IR and neurointerventional radiology were excluded. Respondents working in practices that did not offer the typical spectrum of imaging services (e.g. only performing breast imaging) were also excluded. Questionnaire 2 (Q2) was completed by radiologists who graduated from registrar training within South Africa in the last 15 years. This broad time frame was used for inclusion to ensure that an adequate number of responses would be collected.

In 2023, the RSSA estimated that approximately 500 radiologists were employed in the private sector in South Africa, with 200 radiology registrars in training. Approximately 85% and 90% of these populations were RSSA members, respectively. Convenience sampling was used, and participation was offered to all RSSA members.

### Content of the questionnaires

The questionnaires included a list of IR procedures, developed from a Delphi survey conducted by J. Janse van Rensburg (Table 1). This study aimed to define the core knowledge and skills expected of radiology graduates at the University of the Free State.^3^ As this list represented a consensus-based set of core procedures in the South African setting, it was used in full without modification.

**TABLE 1:** List of interventional radiology procedures.

Variable	Procedure
Non-Vascular	Renal biopsy
	Percutaneous nephrostomy
	Tunnelled dialysis catheter placement
	Percutaneous transhepatic cholangiography (PTC)
	Biliary stent/drain placement
	Percutaneous cholecystostomy
	Liver and pancreas biopsy
	Intra-abdominal drain placement
	Image-guided lumbar puncture (LP) and myelography
Vascular	Aortogram
	Peripheral arteriography
	Mesenteric angiography
	Percutaneous transluminal angioplasty (PTA)
	Peripheral vascular stenting
	IVC filter placement
	Central line placement
	Embolism retrieval
	Thrombolysis
	Renal embolization
	GI bleed-related embolisation
	Uterine artery embolisation
	Cerebral angiography
	Bronchial artery embolisation

*Source:* Janse van Rensburg J. A competency-based continuous assessment programme as part of a revised curriculum for postgraduate radiology training at the University of the Free State [Ph.D]. Bloemfontein: University of the Free State; 2015

IVC, inferior vena cava; GI, gastrointestinal.

In the current study, private-sector radiologists without IR subspecialist training were asked in Q1 about which procedures they performed in their practice and the frequency, level of self-reported competence and source of training for these procedures. Recent graduates were asked in Q2 about their exposure to IR during their registrar training and their level of self-reported competence in the procedures they were exposed to.

The list obtained from J. Janse van Rensburg’s research did not include certain procedures such as pleural and ascitic taps or joint injections for pain relief and arthrography. In J. Janse van Rensburg’s original research, these procedures were included under the skills needed in other modalities or systems (e.g. chest imaging). While still important practical skills in the broader scope of what a newly graduated radiologist should have attained, these procedures were not considered part of the core discipline of IR and were thus excluded from the study.

The questionnaires were developed with neutral wording to avoid posing leading questions. For questions specifically addressing respondents’ perceptions of their registrar training, it was explicitly stated that the questionnaire would not collect any information regarding where the respondent trained. Limited identifying information was collected to improve response rates and encourage candid responses: year of birth, year of qualification and the province where the respondent currently worked. The questionnaires were not formally validated for construct or criterion validity, as is typical for descriptive, procedure-specific self-reported surveys of this kind. Content validity was supported by the use of a Delphi-derived procedure list. A pilot study of ten participants was performed prior to full data collection to ensure the questionnaires were clear and aligned with study objectives. As no changes were made to the questionnaires following the pilot study, these 10 responses were retained and used in the final analysis.

### Data analysis

Categorical data were summarised using frequencies and percentages. Continuous variables were described using the median, as there were no normally distributed variables. Because of the limited sample sizes, confidence intervals could not be meaningfully calculated for these data. Once data collection was complete, the corresponding author cleaned the data, and records with incomplete information were excluded.

### Ethical considerations

An application for full ethical approval was made to the Health Sciences Research Ethics Committee at the University of the Free State, and ethics approval was received on 15 August 2023. The ethics approval number is UFS-HSD2023/0666/2908. All procedures performed in studies involving human participants were in accordance with the ethical standards of the institutional and/or national research committee and with the 1964 Helsinki Declaration and its later amendments or comparable ethical standards.

Participants were presented with the participant information leaflet and consent form before completing the questionnaires. Participants were requested to read all the information thoroughly to make an informed decision. Completion of the questionnaires implied informed consent was given, as per ethical approval. Confidentiality was maintained as only minimal identifying information was collected, and participants were not asked to provide any contact details. Collected data were fed directly from the online questionnaires into the research database, which was protected behind the University of the Free State firewalls. Access to the research database was password-protected.

## Results

A total of 46 respondents completed more than 90% of the questions in Q1 (Table 2a). Based on estimations from the RSSA, this represents 46/425 (11%) of radiologists who are RSSA members. In all, 2/46 (4%) respondents currently worked in both the public and private sectors. The majority of respondents worked in the Western Cape (18/46, 39%) and Gauteng (12/46, 26%).

**TABLE 2a:** Questionnaire 1: Radiologists in private practice - Demographics.

Number of respondents	Age (years)		Years since qualification		Years working in the private sector							
					< 5		5–10		10–20		> 20	
	Range	Median	Range	Median	*N*	%	*N*	%	*N*	%	*N*	%
46	34–66	45	1–34	8	15	33	12	26	7	15	12	26

A total of 29 responses were received for Q2 (Table 2b). Three were excluded due to incomplete data, where less than 50% of the questionnaire was completed. Responses with this level of incompleteness were considered unlikely to contribute meaningful data and risked introducing inconsistencies into the dataset. The remaining 26/29 questionnaires were fully completed. Two of the remaining 26 respondents who had qualified between 11 and 15 years prior were excluded from analysis post hoc. This exclusion reflected both the small number of responses in this interval, which precluded meaningful comparison, and the aim of narrowing the time frame since graduation to better represent current registrar training conditions. The remaining 24/26 responses from radiologists who had graduated within the preceding 10 years were retained for analysis. These 24 responses represent 24/400 (6%) of the number of radiology registrars who graduated in the past 10 years, as estimated by the RSSA. The respondents’ demographics are presented in Table 2a and Table 2b.

**TABLE 2b:** Questionnaire 2: Recent radiology graduates - Demographics.

Number of respondents	Age (years)		Years since qualification	
	Range	Median	Range	Median
24	34–57	39	1–9	3

### Private-practice radiologists

Top procedures performed by private-sector radiologists are shown in Table 3. All procedures were performed by at least one respondent, with respondents performing between 0 and 20 procedures (median = 4). Two of 46 (4%) respondents performed no procedures.

**TABLE 3:** Top procedures performed by private-practice radiologists.

Procedure	Total respondents	Respondents performing the procedure	Percentage of total respondents (%)
Abdominal drains	46	42	91
Lumbar puncture or myelography	46	41	91
Liver or pancreas biopsy	46	37	80
Renal biopsy	46	29	63
Percutaneous cholecystostomy	46	15	33
Percutaneous nephrostomy	46	15	33
Biliary stenting and drainage	46	13	28
Percutaneous transhepatic cholangiography	46	10	22
Renal embolisation	46	6	13
Other	46	≤ 5	≤ 11

The frequency of procedures was calculated by dividing the number of respondents who performed a procedure in each frequency category by the total number of respondents. Non-vascular procedures were generally performed more frequently than vascular procedures. The procedures performed four or more times per month by the most respondents were non-vascular, categorised as most frequent procedures (MFPs). A further four non-vascular procedures were identified as the next category of less frequent procedures (LFPs) (Table 4). Responses relating to vascular procedures are elaborated on below.

**TABLE 4:** Frequency and skill of non-vascular procedures performed by private-practice radiologists.

Frequency categories	Non-vascular procedures	Performed ≥ 4 times per month		Performed < 12 times per year		Performed independently	
		*n/N*	%	*n/N*	%	*n/N*	%
Most frequent procedures (MFP’s)	LP and myelography	11/46	24	14/46	30	41/41	100
	Liver and pancreas biopsies	7/46	15	19/46	41	37/37	100
	Intra-abdominal drains	7/46	15	18/46	39	41/42	98
	Renal biopsies	5/46	11	13/46	28	25/29	86
Less frequent procedures (LFP’s)	Percutaneous nephrostomy	2/46	4	11/46	24	12/15	80
	PTC	2/46	4	8/46	17	8/10	80
	Biliary stent/drain	1/46	2	11/46	24	11/13	85
	Cholecystostomy	1/46	2	11/46	24	15/15	100
-	Tunnelled dialysis catheters	1/46	2	0/46	0	1/1	100

LP, lumbar puncture; PTC, percutaneous transhepatic cholangiography.

Based on the procedures respondents performed, they were asked at what level of competency they felt they could perform them. Most respondents who performed the four MFPs reported that they could perform them independently (Table 4). Of the procedures they could perform independently, participants were then asked where they had acquired the skills to perform these procedures independently. Respondents learnt to perform the four MFPs while working in the private sector in 28%–56% of cases (Figure 1).

**FIGURE 1:**
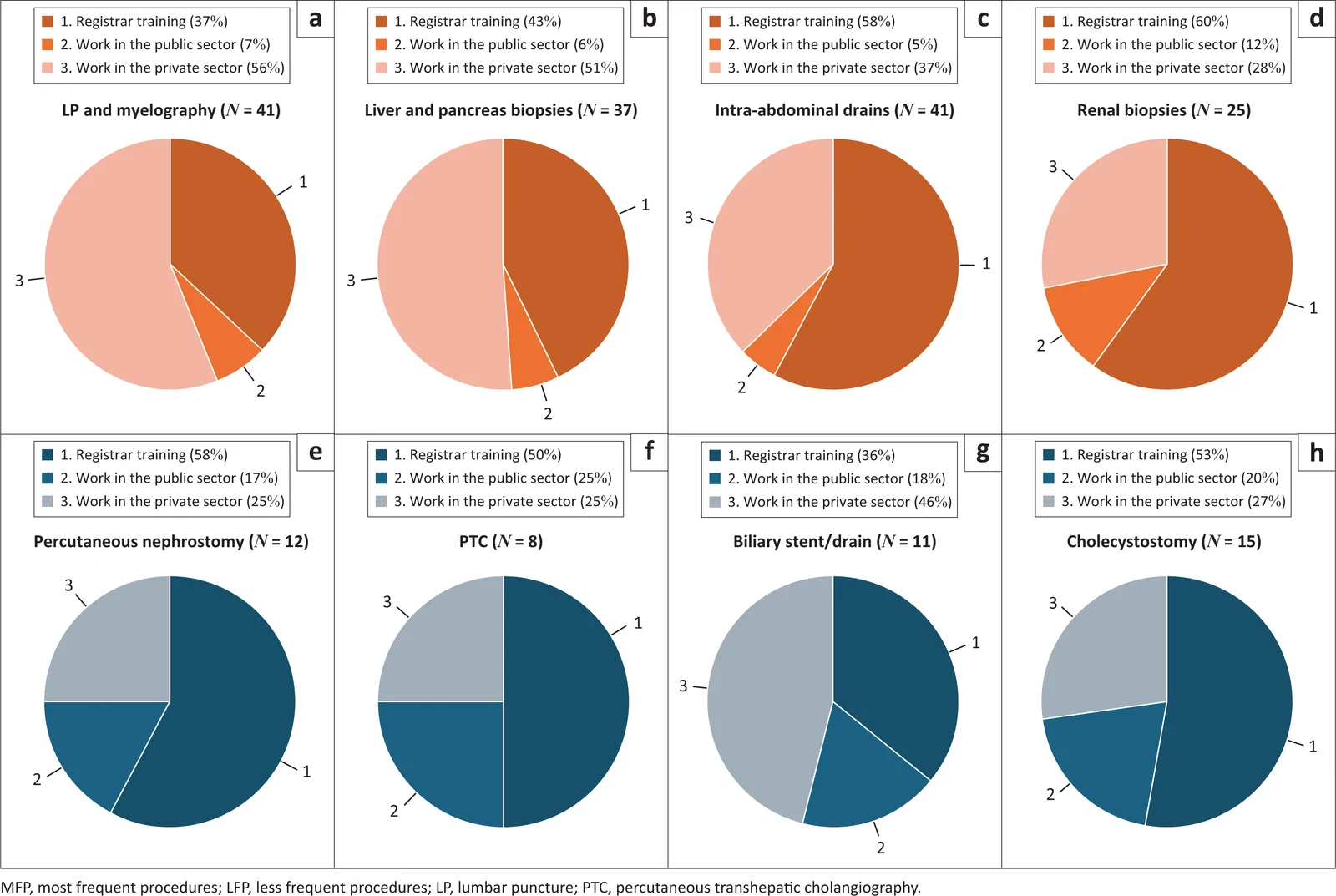
Origin of procedural skills obtained by private-practice radiologists to perform the most frequent procedures: (a) LP and myelography, (b) liver and pancreas biopsies, (c) intra-abdominal drains, (d) renal biopsies, and less frequent procedures, (e) percutaneous nephrostomy, (f) PTC, (g) biliary stent/drain and (h) cholecystostomy.

Of all the procedures performed by the respondents, 225/251 (90%) were performed independently. Among all procedures performed independently by respondents, a comparable proportion reported having acquired the necessary experience during registrar training (103/225, 46%) and work in the private sector (100/225, 44%). An additional 22/225 (10%) of the independently performed procedures were learnt during consultant work in the public sector. The non-vascular procedures performed by most respondents (> 50%) and learnt during registrar training included abdominal drains, renal biopsies, percutaneous nephrostomy and cholecystostomy (4/9 procedures, 44%). Only a small number of respondents were able to perform vascular procedures independently. Overall, there were fewer vascular procedures, which the majority of respondents performing them reported learning during registrar training (4/14, 29%). These included renal embolisation, peripheral arteriography, cerebral angiography and aortography.

Vascular procedures accounted for the majority (14/23, 61%) of procedures included in the study’s procedure list, but were overall infrequently performed (Table 5). Most were performed independently by 4/46 radiologists (9%). Most of these respondents were in their 60s (3/4) and had more than 20 years of experience in the private sector (3/4). These four respondents performed 5–13 of the 14 vascular procedures, mostly independently. The source of skills was variable, but 40%–100% of procedures per participant were learnt in the private sector (Table 6). Most of these respondents (3/4) thought their registrar training was adequate. Five additional respondents performed vascular procedures and had worked in the private sector for less than 10 years. Most did not perform vascular procedures independently, except two who performed renal embolisation and one who performed bronchial artery embolisation independently.

**TABLE 5:** Frequency of vascular procedures performed in private practice.

Vascular procedures	≥ 4 times per month		< 12 times per year	
	Number of radiologists performing this procedure	Frequency (% of all respondents)	Number of radiologists performing this procedure	Frequency (% of all respondents)
Aortogram	2	4	1	2
Peripheral arteriography	2	4	2	4
Mesenteric angiography	1	2	2	4
PTA	1	2	1	2
Peripheral vascular stenting	1	2	2	4
IVC filter placement	0	0	3	7
Central line placement	2	4	0	0
Embolism retrieval	0	0	3	7
Thrombolysis	0	0	4	9
Renal embolisation	0	0	6	13
GI bleed related embolisation	0	0	3	7
Uterine artery embolisation	0	0	0	0
Cerebral angiography	0	0	3	7
Bronchial artery embolisation	0	0	3	7

PTA, percutaneous transluminal angioplasty; IVC, inferior vena cava; GI, gastrointestinal.

**TABLE 6:** Radiologists performing most of the vascular procedures independently in private practice.

‘Vascular’ participant	Number of procedures performed per radiologist (*n/N*)	Performed independently		Where the skills were obtained to perform vascular procedures independently	Procedures (*n*)
		*n/N*	%		
A	5/14	5/5	100	Registrar training	3
				Work in the public sector	0
				Work in the private sector	2
B	8/14	6/8	75	Registrar training	1
				Work in the public sector	2
				Work in the private sector	3
C	13/14	11/13	85	Registrar training	0
				Work in the public sector	0
				Work in the private sector	11
D	9/14	9/9	100	Registrar training	4
				Work in the public sector	0
				Work in the private sector	5

Private-practice radiologists were also asked if they felt registrar training prepared them for private-sector work. Forty-three per cent of respondents (20/46) said they received enough exposure to IR. The number of respondents who would have wanted more training during registrar time in the four MFPs is presented in Table 7.

**TABLE 7:** Private-practice radiologists who would have wanted more training in the most frequent procedures during registrar time.

Procedure	Number†	%
Renal biopsies	20/45	44
Liver and pancreas biopsies	20/45	44
Intra-abdominal drains	13/45	29
LP and myelography	16/45	36

† , One respondent didn’t answer this question.

LP, lumbar puncture.

### Recent radiology graduates

Most respondents had a formal IR rotation (17/24, 71%), and just less than half (11/24, 46%) thought their IR training was sufficient to prepare them for work in the private sector. The commonest reasons for feeling training was insufficient were: ‘Too few cases of certain procedures’ (9/13, 69%) and ‘Not a wide enough range of procedures’ (11/13, 85%). The top procedures of insufficient cases were percutaneous nephrostomy (7/9, 78%) and percutaneous cholecystostomy (7/9, 78%).

Respondents reported receiving training in 3–19 of the total 23 procedures (median = 9). The top procedures respondents were exposed to were abdominal drains (23/24, 96%), liver or pancreas biopsy (23/24, 96%) and percutaneous transhepatic cholangiography (PTC) (20/24, 83%) (Table 8). Most respondents trained in abdominal drains (17/23, 74%) and liver or pancreas biopsy (15/23, 65%) could perform these procedures independently solely based on registrar training. However, the level of self-reported competence for PTCs was considerably lower (7/20, 35%).

**TABLE 8:** Recent graduates’ exposure to non-vascular procedures during registrar training and level of self-reported competency.

Procedure	Non-vascular procedures	Exposed to the procedure		Can perform it independently	
		*n/N*	%	*n/N*	%
Most frequent procedures (MFP’s)	Liver and pancreas biopsies	23/24	96	15/23	65
	Intra-abdominal drains	23/24	96	17/23	74
	Renal biopsies	18/24	75	11/18	61
	LP and myelography	14/24	58	13/14	93
Less frequent procedures (LFP’s)	PTC	20/24	83	7/20	35
	Percutaneous nephrostomy	19/24	79	9/19	47
	Biliary stent/drain	14/24	58	5/14	36
	Cholecystostomy	14/24	58	11/14	79
-	Tunnelled dialysis catheters	4/24	17	1/4	25

PTC, percutaneous transhepatic cholangiography; LP, lumbar puncture.

Less than 50% of the respondents were exposed to any form of vascular procedure. The top procedures they were exposed to were aortograms (11/24, 46%), peripheral arteriography (11/24, 46%) and bronchial artery embolisation (9/24, 38%). Less than half of these respondents could perform these procedures independently (Table 9).

**TABLE 9:** Recent graduates’ exposure to vascular procedures during registrar training and level of self-reported competency.

Vascular procedures	Exposed to the procedure		Can perform independently if exposed to	
	*n/N*	%	*n/N*	%
Aortogram	11/24	46	4/11	36
Peripheral arteriography	11/24	46	4/11	36
Bronchial artery embolisation	9/24	38	2/9	22
Central line placement	7/24	29	5/7	71
Cerebral angiography	7/24	29	0/7	0
Renal embolization	6/24	25	3/6	50
GI bleed related embolisation	6/24	25	2/6	33
Mesenteric angiography	5/24	21	2/5	40
Uterine artery embolisation	5/24	21	1/5	20
IVC filter placement	3/24	13	0/3	0
Peripheral vascular stenting	1/24	4	0/1	0
Thrombolysis	1/24	4	0/1	0
PTA	0/24	0	0/0	0
Embolism retrieval	0/24	0	0/0	0

PTA, percutaneous transluminal angioplasty; IVC, inferior vena cava; GI, gastrointestinal.

### Potential confounding factors

Variability among private practices may have influenced responses, including differences between larger and smaller urban settings, referral patterns and local availability of resources. For example, practices in areas served by vascular surgeons may have had lower demand for vascular procedures, and access to theatre time, equipment or support staff may have influenced which procedures individual radiologists were able to perform, regardless of training. Where subspecialist interventional radiologists were present within a practice, generalist colleagues may also have been expected to perform fewer complex procedures. Credentialing requirements for certain procedures may similarly have influenced what individual radiologists performed in practice. While radiologists with interventional subspecialist training were excluded from this study, their generalist colleagues in the same practices were included, which may have reduced the likelihood of complex procedures being reported.

Similar heterogeneity likely exists among recent graduates, due to differences in university training, equipment availability and IR expertise. As responses were self-reported, discrepancies may exist between perceived and actual competence, with some respondents potentially overestimating or underestimating their abilities.

## Discussion

Interventional radiology is a rapidly expanding and evolving specialty. In many countries, distinct training pathways exist for radiologists who intend to perform interventional procedures. However, in South Africa, there are currently only two training centres offering formal subspecialisation in IR, one of which is in abeyance. Consequently, most diagnostic radiologists completing general training are expected to perform at least some interventional procedures in private practice, which is the predominant employment setting for newly qualified radiologists in South Africa.

### Most frequently performed procedures in private practice

It is well described in the literature that procedures such as percutaneous drain placement and liver biopsies have advantages over open surgical procedures.^4,5,6^ These and other non-vascular procedures that the largest number of radiologists performed most frequently, were mostly performed independently (Table 4). However, a considerable number learnt to perform these procedures independently while working in the private sector (Figure 1). This suggests that registrar training during the period these radiologists qualified may not have fully prepared them for the expectations of private practice in terms of IR procedures. Notably, 20/46 (43%) respondents did not state a desire for more exposure to IR during registrar training. A similar number of radiologists would have wanted more training in the four MFPs, except for abdominal drains (Table 7). These data suggest that retrospectively, private radiologists were comfortable with mastering specific skills during consultant work in the public sector or after entering the private sector. There is possibly an expectation from private practices that newly qualified radiologists will often need further training once employed.

A considerable amount (10/24, 42%) of the recent graduates had not been exposed to the procedure performed most frequently in private practice (lumbar puncture [LP] and myelography) during their registrar training. However, most recent graduates who were exposed to LP and myelography as registrars could perform them independently (93%, Table 8). Exposure during registrar training was higher in the other three MFPs in private settings, with moderate levels of self-reported competence (61%–74%, Table 8). Recent graduates may expect that they will master the skills needed for private-practice work while working as a public-sector consultant or after entering the private sector, similar to the suspected experiences of private-practice radiologists, as described above.

Compared to recent graduates (61%–93%), private-practice radiologists (43%–60%) appeared less likely to have been able to perform the MFPs independently after registrar training, except for renal biopsies. This could be due to a generational gap between the two groups (Figure 1 and Table 8). For the private-practice radiologists, the median year of birth and year of qualification are earlier than those of the recent graduates group (Table 2a and Table 2b). The increased recognition of the value of interventional procedures and the greater availability of facilities and consultants experienced in IR, likely contributed to differences in training experiences between the two groups.

### Less frequent procedures performed in private practice

Biliary procedures and percutaneous nephrostomy are known in the literature to have benefits over open surgical procedures.^7,8,9^ These procedures emerged in the data as a category of non-vascular procedures performed less frequently by most private-practice respondents (Table 4).^7,8,9^ Fewer than 5% of respondents reported performing these four procedures with high frequency (at least four times per month). They were, however, performed by between 8/46 (17%) and 11/46 (24%) of respondents less than 12 times per year (Table 4). Of note, there were relatively high rates of exposure to these procedures during registrar training in the recent graduates group (58%–83%, Table 8).

Private-practice radiologists who perform PTCs, biliary stents or drains and percutaneous nephrostomies appear to have had similar levels of experience attained during registrar training as recent graduates (Figure 1 and Table 8). Between 36% and 58% of private radiologists could perform these procedures independently following registrar training, compared to 36% to 47% of recent graduates. Percutaneous cholecystostomies had higher levels of self-reported competence after registrar training in recent graduates (11/14, 79%) than in private radiologists (8/15, 53%), but this equalised after acknowledging skills learnt by private radiologists in the public sector as consultants (3/15, 20%). Overall, a larger proportion of private radiologists improved their skills in the public sector for these LFPs (17%–25%), compared to the MFPs (5%–12%). In summary, these findings suggest that graduates with the skills or interest to perform these LFPs are preferentially selected to perform them in the private-practice setting.

### Vascular procedures

Vascular IR procedures have been documented in the literature to have advantages over open surgery in specific clinical settings.^10,11^ A few private-practice radiologists in the late stages of their careers performed most of the vascular procedures, nearly all of them independently (Table 6). Recent graduates had a relatively low level of exposure to vascular procedures, with even lower rates of ability to perform them independently (Table 9).

As has been mentioned above, there may be an expectation from registrars, radiologists and private practices that new graduates will often not have the skills to start performing interventional procedures soon after registrar training. This seems even more prevalent for vascular procedures. Vascular procedures, especially venous procedures, are more technical and have significant complication rates.^12^ This is likely to make recent graduates hesitant to perform them without consultant supervision. Private practices may also be reluctant to expect younger radiologists to perform these procedures due to concerns regarding litigation. Vascular procedures are often considered more complicated and are often performed by subspecialist interventional radiologists. This is likely why IR is a subspeciality on its own in many other countries. Certain procedures, such as aortograms and peripheral angiography, have also been largely replaced by CT angiography due to superior acquisition speed, wider availability and cost-effectiveness. In many private- and public-sector settings, vascular surgeons have started performing a large proportion of these vascular procedures. This is likely to limit registrar exposure to vascular procedures in the public sector and limit the demand for these procedures in private radiology practices.

### Interventional radiology procedures in the diagnostic radiology curricula

National or region-wide diagnostic radiology curricula are mostly found in high-income countries. Curricula from several English-speaking high-income countries and the European region were assessed for the IR procedures in which new graduates were expected to be proficient. The UK, USA, Canadian, European and Australian and New Zealand regulatory bodies all included three of the four MFPs in the private sector.^13,14,15,16,17^ These included image-guided biopsies and basic drainage procedures. Only the Royal College of Physicians and Surgeons of Canada included LP as one of their required IR procedures.^14^ A review of the available literature yielded no nationally adopted curricula on the African continent outside of South Africa. The Colleges of Medicine do have a published curriculum for diagnostic radiology, but this currently does not include a list of IR procedures that graduates are expected to be proficient in.^1^ In terms of the LFPs described above, only PTC was mentioned as a required procedure in diagnostic radiology training in Europe.^16^ Vascular procedures were not uniformly adopted by the curricula reviewed. European, as well as the Australian and New Zealand regulatory bodies included ‘basic angiography’ as a required skill, while the UK and USA included ‘vascular access’ and ‘venography’.^13,15,16,17^

### The scope of interventional radiology in South Africa

Interventional radiology as a subspecialty encompasses a much larger scope of procedures than what is usually expected of a general radiologist entering private practice. The current study deliberately used private-practice expectations rather than the full breadth of IR subspecialty practice as the comparative standard, since this reflects the minimum level of independent procedural competency required of the majority of graduates who enter private practice without additional IR training.

However, in higher-income countries IR services routinely extend to vascular and venous interventions, interventional oncology and a growing range of embolisation-based treatments for benign conditions such as uterine fibroids and benign prostatic hyperplasia. The ongoing expansion of the field of IR reflects not only technical capacity, but also the evolution of IR into a specialty with its own outpatient service and referral system. As such, there is room for much expansion of the already available IR services in the private sector in South Africa. For this to be achievable, consideration of training programmes for radiology registrars and the further development of IR fellowship programmes in South Africa are needed.

Another point of consideration is whether expectations should differ between registrars intending to enter private practice to only occasionally perform minor IR procedures, and those pursuing a career in IR. For the former, private-practice expectations may reasonably represent the target competency level. For the latter, however, this same standard should perhaps represent a minimum requirement rather than an endpoint, given that career interventional radiologists require considerably more advanced skills than general practice demands. This distinction is represented in the Cardiovascular and Interventional Radiological Society of Europe curriculum, Third Edition published in 2023, which explicitly differentiates training pathways and expected competency levels according to trainees’ subspecialty intent.^18^ Interventional radiology is also a distinct speciality in the USA, separate from diagnostic radiology since 2012.^19^

### Study limitations and implications

Procedural frequency, as reported in this study, reflects current practice patterns among private-practice radiologists rather than a defined expectation of competence for new graduates. This distinction is shaped by several practice-level factors, including case mix, regional referral patterns, the availability of subspecialist interventional radiologists, hospital resources and credentialing requirements. As such, procedures performed infrequently in this study should not necessarily be interpreted as falling outside the expected skill set of a competent graduate.

Response rates for both questionnaires were low. Based on estimations of the target populations, response rates were 11% for private radiologists (Q1) and 6% for recent graduates (Q2). This is often the case with many email-based surveys,^20^ but represents a significant limitation of the study. Such low response rates raise the possibility of non-response bias, where respondents who chose to participate may differ significantly from those who did not. This limits generalisability and external validity of the findings. Low participation rates of recent graduates may also be attributed to factors such as emigration, only performing locums, and eligibility for both questionnaires. Dual eligibility may have resulted in participants completing the first questionnaire but not the second. Additionally, the long list of procedures in the questionnaire may have contributed to survey fatigue.

Selection bias may have occurred due to the voluntary nature of participation, favouring responses from more engaged and motivated individuals. The reliance on self-reported data introduces the possibility of recall bias, particularly among respondents who completed their training many years prior. The exclusion of qualified interventional radiologists likely resulted in the omission of insights into interventional services offered in the private sector. Including their perspectives may have helped clarify specific trends observed in the data. Social desirability bias may have further influenced respondents to report training experiences or procedural involvement in a more favourable light. As the findings reflect self-reported competence rather than objectively assessed skill, they should be interpreted as preliminary.

Although the sample sizes were small, the findings of this study may aid educational institutions in developing learning outcomes for radiology registrars. This emphasises the need for a national curriculum to standardise the knowledge and skills expected of graduates. Further research into future employment settings is also likely to be valuable for registrars during their training.

## Conclusion

In this exploratory study, the procedures most frequently performed in the private sector by non-IR subspecialist radiologists appear to be the best represented IR skills developed by recent graduates during training, with moderate to high levels of self-reported competence. Comparable findings were observed for less frequently performed procedures within private practice. In contrast, vascular interventional procedures performed by non-subspecialists are infrequent and typically carried out by a small subset of radiologists who primarily acquire these skills through private-sector experience. This pattern is reflected in the relatively limited exposure to vascular interventions reported by recent graduates. An avenue for future research involves quantifying the number of IR subspecialists practising in the private sector and assessing their contribution to the overall procedural workload.

This study underscores the need for a clearly defined curriculum in South Africa, delineating the core IR skills that radiology graduates should possess. Such a framework will enable educational institutions to ensure appropriate training coverage and facilitate the restructuring and realignment of radiology training programs. This is especially important in light of the ongoing expansion and evolution of the field of IR, with the scope of practice in the private sector likely to expand and diversify. Further research and engagement with regulatory bodies in South Africa will be important for registrar training programmes to maintain the pace with this ongoing evolution.
